# The association between child maltreatment and mental disorders in the Australian Child Maltreatment Study

**DOI:** 10.5694/mja2.51870

**Published:** 2023-04-02

**Authors:** James G Scott, Eva Malacova, Ben Mathews, Divna M Haslam, Rosana Pacella, Daryl J Higgins, Franziska Meinck, Michael P Dunne, David Finkelhor, Holly E Erskine, David M Lawrence, Hannah J Thomas

**Affiliations:** ^1^ Child Health Research Centre, the University of Queensland Brisbane QLD; ^2^ QIMR Berghofer Medical Research Institute Brisbane QLD; ^3^ Queensland University of Technology Brisbane QLD; ^4^ Bloomberg School of Public Health Johns Hopkins University Baltimore MD United States of America; ^5^ The University of Queensland Brisbane QLD; ^6^ Institute for Lifecourse Development University of Greenwich London United Kingdom; ^7^ Institute of Child Protection Studies Australian Catholic University Melbourne VIC; ^8^ University of Edinburgh Edinburgh United Kingdom; ^9^ University of the Witwatersrand Johannesburg Johannesburg South Africa; ^10^ Institute for Community Health Research Hue University Hue City Vietnam; ^11^ Crimes against Children Research Center University of New Hampshire Durham NH United States of America; ^12^ Queensland Centre for Mental Health Research Brisbane QLD; ^13^ Curtin University Perth WA

**Keywords:** Mental disorders, Mental health policy, Epidemiology, Child abuse, Child welfare

## Abstract

**Objectives:**

To examine the associations between experiences of child maltreatment and mental disorders in the Australian population.

**Design:**

Population‐representative survey conducted by computer‐assisted telephone interviewing.

**Setting, participants:**

Australian residents aged 16 years and older.

**Main outcome measures:**

Mental disorder diagnoses of lifetime major depressive disorder, current alcohol use disorder (mild, moderate and severe), current generalised anxiety disorder and current post‐traumatic stress disorder.

**Results:**

More than one in three Australians (3606/8503 surveyed participants; 38.0%; 95% CI, 36.7–39.3%) met the diagnostic criteria for a mental disorder. The prevalence of mental disorders in non‐maltreated participants was 21.6% (95% CI, 19.9–23.3%; *n* = 851). This increased to 36.2% (95% CI, 33.5–38.9%; *n* = 764) for those who experienced a single type of maltreatment and 54.8% (95% CI, 52.6–56.9%; *n* = 1991) for participants who experienced multi‐type maltreatment. Compared with non‐maltreated Australians, maltreated participants had about three times the odds of any mental disorder (odds ratio [OR], 2.82; 95% CI, 2.47–3.22), generalised anxiety disorder (OR, 3.14; 95% CI, 2.48–3.97), major depressive disorder (OR, 3.19; 95% CI, 2.68–3.80) and severe alcohol use disorder (OR, 2.62; 95% CI, 1.83–3.76), and almost five times the odds of post‐traumatic stress disorder (OR, 4.60; 95% CI, 3.00–7.07). Associations between experiences of child maltreatment and mental disorders were strongest for sexual abuse, emotional abuse and multi‐type maltreatment. The strength of the associations did not differ by gender. Adjustment for childhood and current financial hardship and for current socio‐economic status did not significantly attenuate the associations.

**Conclusions:**

Mental disorders are significantly more likely to occur in individuals who experience child maltreatment, particularly multi‐type maltreatment. Prevention of child maltreatment provides an opportunity to substantially reduce the prevalence of mental illness and improve the health of the Australian population.

**The known:** Child maltreatment is associated with increased risk of mental disorders.

**The new:** To our knowledge, this is the most comprehensive assessment of the relationship between childhood maltreatment and mental disorders in Australia to date. Children who are emotionally abused, sexually abused or exposed to multi‐type maltreatment are at particularly high risk of mental disorders. The prevalence of common mental disorders is relatively low in people who were not maltreated during childhood. Associations between child maltreatment and mental disorders remained significant after adjustment for sociodemographic factors.

**The implications:** Prevention of child maltreatment is critical to achieve a meaningful reduction in the prevalence of mental disorders.

In Australia, mental disorders and substance use disorders are the leading causes of non‐fatal disease burden, accounting for almost one‐quarter of years lived with disability.^1^ The National Survey of Mental Health and Wellbeing, which collected data between December 2020 and July 2021, showed that more than two in five Australians (43.7%) had experienced a mental disorder during their lifetime, with major depressive disorder (MDD), anxiety disorders, post‐traumatic stress disorder (PTSD) and alcohol use disorders (AUDs) being highly prevalent.^2^ Addressing modifiable risk factors for mental disorders may reduce their prevalence and associated morbidity.^3^

Maltreatment in childhood is a well established risk factor for mental illness across the lifespan.^4^ Although studies have shown that people who experience child maltreatment — defined as physical abuse, emotional abuse, sexual abuse, neglect or exposure to domestic violence before 18 years of age — have a 2–3‐fold increased risk of mental health problems,^4^, ^5^ research in this field has common limitations. Assessments of child maltreatment have used a wide range of definitions and are typically restricted to a limited number of maltreatment types. Further, assessments of mental illness commonly involve symptom scales rather than diagnostic instruments.^6^ These limitations result in variability in the strength of reported associations. In addition, although children often experience multi‐type maltreatment,^7^ few studies examining associations between one type of maltreatment and mental illness adjust for other forms of maltreatment,^8^ potentially leading to an overestimation of the strength of associations. Similarly, most studies do not assess the association between experiences of multi‐type child maltreatment and mental disorders.

To our knowledge, the Australian Child Maltreatment Study (ACMS) is the first national survey to measure both the prevalence of all five forms of child maltreatment and diagnoses of mental disorders in Australia's population aged 16 years and older.^9^ A detailed explanation of our approach to the conceptual models of each maltreatment type in the ACMS is outlined in our protocol article.^9^ Our operationalisation of these maltreatment types (including their integration within survey items) is explained in our methodology article.^10^ In this study, we aimed to estimate the strength of the associations between each form of maltreatment and each diagnosis of a common mental disorder in the Australian community. We also aimed to provide national population estimates for common mental disorders.

## Methods

### Participants

Full details of the methods for the ACMS are described elsewhere in this supplement.^10^ In brief, a random sample of Australians aged 16 years and older was recruited via mobile phone by random digit dialling, following an advance text message inviting participation. Data were collected by computer‐assisted telephone interviewing, which was conducted by trained lay interviewers. Comparison with Australian census and survey data showed that the weighted data were representative of the Australian population. Ethics clearance was obtained from the Human Research Ethics Committee of Queensland University of Technology (approval number 1900000477) and all participants gave informed consent.

### Measures

Child maltreatment was assessed using 16 screener items from the Juvenile Victimisation Questionnaire‐R2 adapted version (Australian Child Maltreatment Study). These behaviourally specific questions elicited a dichotomous yes‐or‐no response, identifying whether the participant had experienced any subdomain of each maltreatment type (physical abuse, two subdomains; sexual abuse, four subdomains; emotional abuse, three subdomains; neglect, three subdomains; and exposure to domestic violence, four subdomains [Supporting Information, table 1]). Physical abuse, sexual abuse and exposure to domestic violence were based on positive endorsement of any of the screener items for these maltreatment types, regardless of how many times the experience happened. Neglect and emotional abuse were only counted as positively endorsed if the experiences were reported to have occurred over a period of weeks, months or years.^11^ In the ACMS, neglect refers to environmental neglect, physical neglect or medical neglect. Emotional unavailability was included in emotional abuse.

In the Diagnostic and Statistical Manual of Mental Disorders, 5th edition (DSM‐5), diagnoses of generalised anxiety disorder (GAD) (current), PTSD (current), AUD (current; mild, moderate and severe) and MDD (lifetime) were established using modules of the Mini International Neuropsychiatric Interview (MINI), version 7.0.2. Measurement of lifetime prevalence was limited to MDD to reduce participant burden. The MINI is a short, structured diagnostic instrument that is valid and reliable;^12^, ^13^ it is administered by trained lay interviewers and is widely used in epidemiological surveys of mental health. Financial hardship during childhood was assessed by asking “How often did your family experience economic hardship such as finding it difficult to provide food, medical care, or other basic necessities?” People were considered to have experienced childhood financial hardship if their response was “somewhat often” or “very often”. Current financial strain was assessed by asking “In the past 12 months, has there been a time when your household could not meet essential expenses?” Participants were considered to be experiencing current financial strain if their response was “yes”.

### Statistical analysis

Missing data were excluded from the analysis. Survey‐weighted prevalence rates and 95% confidence intervals for mental disorders were calculated using the method of expansion in Taylor series.^14^ Mental disorder prevalence estimates were then compared by experiences of child maltreatment (no maltreatment, any maltreatment, single type maltreatment and multi‐type maltreatment [two or more types]) and by gender.

Survey‐weighted logistic regression models were used to examine the associations between experiences of child maltreatment and diagnosis of each mental disorder. The first model calculated the odds ratios (ORs) and 95% confidence intervals (CIs) for experiences of any child maltreatment compared with no child maltreatment. The second model calculated the odds of each mental disorder with experiences of each of the five types of child maltreatment fitted simultaneously as independent binary (yes/no) variables. This enabled associations between each mental disorder and each type of maltreatment to be adjusted for the experiences of other types of maltreatment. Each model was fitted with two levels of adjustment for other factors. The simple adjusted model accounted for age group and gender, while the fully adjusted model also accounted for child maltreatment, socio‐economic disadvantage (based on postcode of residence and quintiles of the Index of Relative Socio‐Economic Disadvantage [one of the Socio‐Economic Indexes for Areas]),^15^ financial hardship during childhood, and current financial strain.

## Results

In total, 8503 participants completed the survey, and fewer than 1% of data were missing. The survey‐weighted national prevalence estimates for mental disorders in Australian residents aged 16 years and older, in those with and without experiences of child maltreatment, are shown in Box 1. More than one in three participants (38.0%; 95% CI, 36.7–39.3%; *n* = 3606) had any mental disorder, either currently (GAD, AUD, PTSD) or during their lifetime (MDD). The prevalence estimates for lifetime MDD, current GAD and current PTSD were 18.4% (95% CI, 17.4–19.4%; *n* = 1716), 11.7% (95% CI, 10.8–12.5%; *n* = 1148) and 5.3% (95% CI, 4.7–5.9%; *n* = 488), respectively. Mild, moderate and severe current AUDs were diagnosed in 10.7% (95% CI, 9.9–11.5%; *n* = 1058), 4.7% (95% CI, 4.2–5.3%; *n* = 486) and 4.6% (95% CI, 4.0–5.1%; *n* = 396) of participants, respectively. There was no significant gender difference in the proportions of participants with at least one type of mental disorder, and men and women reported a similar prevalence of PTSD. Prevalence estimates for mild, moderate and severe AUDs were greater in men, whereas prevalence estimates for GAD and MDD were higher in women (Box 1).

Box 1 **Prevalence of mental disorders in Australians 16 years and older, by experience of child maltreatment (*N* = 8503)**

**Table:** 

	Participants — number; percentage (95% CI)		
	Men (*n* = 4195)	Women (*n* = 4182)	All genders* (*n* = 8503)
**Any mental disorder**			
Total	1699; 38.0% (36.2–39.9%)	1824; 37.7% (35.9–39.5%)	3606; 38.0% (36.7–39.3%)
No child maltreatment	509; 25.0% (22.4–27.5%)	335; 17.7% (15.4–20.1%)	851; 21.6% (19.9–23.3%)
Any child maltreatment	1190; 47.3% (44.8–49.9%)	1489; 48.2% (45.8–50.5%)	2755; 48.0% (46.2–49.7%)
One type of child maltreatment	396; 40.1% (36.2–44.1%)	360; 32.3% (28.6–36.0%)	764; 36.2% (33.5–38.9%)
Two or more types of child maltreatment	794; 52.2% (48.9–55.5%)	1129; 56.4% (53.5–59.3%)	1991; 54.8% (52.6–56.9%)
**Post‐traumatic stress disorder** ^†^			
Total	167; 4.4% (3.6–5.3%)	296; 5.9% (5.1–6.8%)	488; 5.3% (4.7–5.9%)
No child maltreatment	20; 1.3% (0.6–2.0%)	20; 1.2% (0.5–1.9%)	41; 1.3% (0.8–1.7%)
Any child maltreatment	147; 6.7% (5.4–8.0%)	276; 8.4% (7.2–9.7%)	447; 7.8% (6.9–8.7%)
One type of child maltreatment	23; 2.8% (1.4–4.2%)	28; 2.5% (1.2–3.8%)	53; 2.6% (1.7–3.6%)
Two or more types of child maltreatment	124; 9.3% (7.4–11.3%)	248; 11.5% (9.7–13.3%)	394; 10.8% (9.5–12.1%)
**Generalised anxiety disorder** ^†^			
Total	419; 9.8% (8.6–10.9%)	671; 13.0% (11.7–14.2%)	1148; 11.7% (10.8–12.5%)
No child maltreatment	76; 4.1% (2.9–5.3%)	96; 4.5% (3.3–5.7%)	175; 4.3% (3.5–5.2%)
Any child maltreatment	343; 13.8% (12.0–15.5%)	575; 17.4% (15.7–19.2%)	973; 16.1% (14.9–17.3%)
One type of child maltreatment	75; 7.5% (5.3–9.7%)	102; 8.5% (6.4–10.7%)	182; 8.1% (6.5–9.6%)
Two or more types of child maltreatment	268; 18.0% (15.5–20.5%)	473; 22.0% (19.7–24.4%)	791; 20.8% (19.1–22.5%)
**Alcohol use disorder — mild** ^†^			
Total	619; 13.5% (12.2–14.8%)	428; 8.1% (7.1–9.1%)	1058; 10.7% (9.9–11.5%)
No child maltreatment	243; 12.0% (10.1–13.9%)	102; 5.5% (4.1–6.8%)	345; 8.9% (7.7–10.1%)
Any child maltreatment	376; 14.5% (12.7–16.3%)	326; 9.5% (8.2–10.8%)	713; 11.8% (10.7–12.8%)
One type of child maltreatment	163; 16.0% (13.1–18.9%)	86; 7.1% (5.1–9.2%)	251; 11.5% (9.7–13.3%)
Two or more types of child maltreatment	213; 13.5% (11.2–15.7%)	240; 10.7% (9.0–12.4%)	462; 11.9% (10.5–13.2%)
**Alcohol use disorder — moderate** ^†^			
Total	267; 5.7% (4.8–6.6%)	211; 3.8% (3.1–4.5%)	486; 4.7% (4.2–5.3%)
No child maltreatment	76; 3.6% (2.5–4.8%)	36; 1.9% (1.1–2.7%)	112; 2.8% (2.1–3.5%)
Any child maltreatment	191; 7.2% (5.9–8.5%)	175; 4.8% (3.9–5.7%)	374; 5.9% (5.1–6.7%)
One type of child maltreatment	60; 6.1% (4.2–8.1%)	40; 2.6% (1.5–3.7%)	101; 4.3% (3.2–5.4%)
Two or more types of child maltreatment	131; 7.9% (6.2–9.6%)	135; 5.9% (4.6–7.2%)	273; 6.8% (5.8–7.8%)
**Alcohol use disorder — severe** ^†^			
Total	220; 5.5% (4.6–6.4%)	164; 3.6% (2.9–4.3%)	396; 4.6% (4.0–5.1%)
No child maltreatment	43; 2.9% (1.8–3.9%)	19; 0.9% (0.4–1.4%)	62; 1.9% (1.3–2.6%)
Any child maltreatment	177; 7.4% (6.1–8.8%)	145; 5.0% (3.9–6.0%)	334; 6.1% (5.3–7.0%)
One type of child maltreatment	60; 6.2% (4.2–8.2%)	22; 2.8% (1.5–4.2%)	82; 4.5% (3.3–5.7%)
Two or more types of child maltreatment	117; 8.2% (6.4–10.1%)	123; 6.1% (4.7–7.5%)	252; 7.1% (6.0–8.2%)
**Major depressive disorder** ^‡^			
Total	698; 15.4% (14.0–16.7%)	989; 21.2% (19.7–22.8%)	1716; 18.4% (17.4–19.4%)
No child maltreatment	165; 7.4% (5.9–8.8%)	162; 8.9% (7.2–10.6%)	330; 8.1% (7.0–9.2%)
Any child maltreatment	533; 21.1% (19.0–23.1%)	827; 27.7% (25.7–29.8%)	1386; 24.6% (23.2–26.1%)
One type of child maltreatment	149; 14.6% (11.8–17.5%)	211; 19.7% (16.6–22.8%)	363; 17.2% (15.1–19.3%)
Two or more types of child maltreatment	384; 25.4% (22.6–28.3%)	616; 31.9% (29.2–34.6%)	1023; 28.9% (27.0–30.9%)

* Includes participants who identified as gender diverse.

† Current.

‡ Lifetime.

Prevalence estimates varied significantly with experiences of child maltreatment, as evidenced by non‐overlapping CIs (Box 1). The prevalence of mental disorders in non‐maltreated participants was 21.6% (95% CI, 19.9–23.3%; *n* = 851). This increased to 36.2% (95% CI, 33.5–38.9%; *n* = 764) for those who experienced a single type of maltreatment and 54.8% (95% CI, 52.6–56.9%; *n* = 1991) for participants who experienced multi‐type maltreatment. For each type of mental disorder, the prevalence was also significantly greater in those who had experienced any child maltreatment. For example, the prevalence estimates for lifetime MDD in those with and without experiences of child maltreatment were 24.6% (95% CI, 23.2–26.1%; *n* = 1386) and 8.1% (95% CI, 7.0–9.2%; *n* = 330), respectively. Prevalence of PTSD was particularly low in non‐maltreated individuals (1.3%; 95% CI, 0.8–1.7%; *n* = 41) compared with maltreated individuals (7.8%; 95% CI, 6.9–8.7%; *n* = 447). In those who experienced child maltreatment, the gender differences in prevalence were the same as in the total sample, with AUDs being more prevalent in men and GAD and MDD being more prevalent in women. With the exception of mild AUD, a dose–response relationship was observed — those who experienced multi‐type maltreatment had a significantly higher prevalence of each mental disorder compared with those who experienced one type of maltreatment.

The proportions of participants who met the diagnostic criteria for a mental disorder varied by age, gender and experience of maltreatment (Box 2). In all age groups, the proportion of participants with lifetime MDD was much greater in those who reported child maltreatment. With the exception of mild AUD, mental disorders were relatively uncommon (< 10% prevalence) in those aged 45 years and older who reported no experiences of child maltreatment. Exact proportions of people with mental disorders exposed to child maltreatment are reported in Supporting Information, table 2.

Box 2 Proportions of Australians 16 years and older with mental disorders, by age group, gender and experience of child maltreatment*

**Figure:**
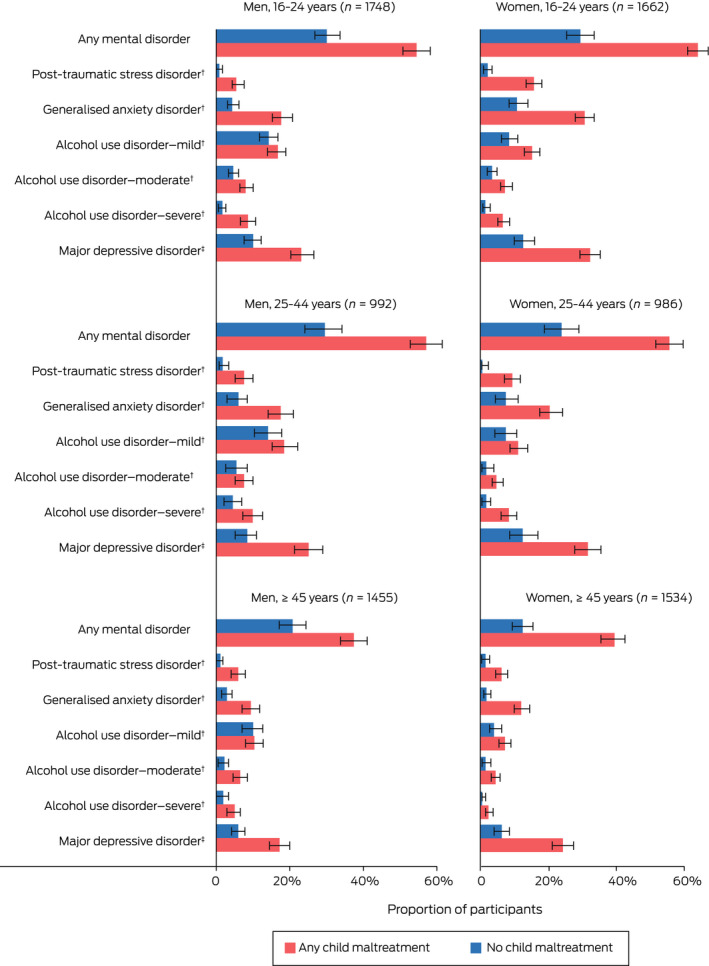
* Bar lengths represent percentage of participants with mental disorders and error bars represent 95% CIs. † Current. ‡ Lifetime.

The simple and fully adjusted odds of having each mental disorder for those who experienced maltreatment relative to those who did not are shown in Box 3. Those who were maltreated had almost three times the odds of meeting the diagnostic criteria for any mental disorder, MDD, GAD and severe AUD when compared with those who had no experience of maltreatment. The likelihood of being diagnosed with PTSD was almost five times higher in those who reported experiences of maltreatment (OR, 4.60; 95% CI, 3.00–7.07) than those who did not. Compared with those with no maltreatment, the odds of having a mental disorder were higher for women than for men among those who experienced maltreatment. Adjustment for childhood or current financial hardship and current socio‐economic status did not have a significant effect on the associations.

Box 3 **Odds ratios for mental disorders in Australians 16 years and older who experienced child maltreatment relative to those who did not**

**Table:** 

	Odds ratio (95% CI) — adjusted for age and gender			Odds ratio (95% CI) — fully adjusted		
	Men*	Women*	Total^†^	Men^‡^	Women^‡^	Total^§^
Any mental disorder	2.62 (2.20–3.12)	4.26 (3.52–5.15)	3.29 (2.89–3.73)	2.28 (1.90–2.72)	3.65 (2.99–4.45)	2.82 (2.47–3.22)
Post‐traumatic stress disorder^¶^	5.18 (2.88–9.31)	7.33 (4.00–13.40)	6.21 (4.10–9.40)	3.77 (2.07–6.86)	5.43 (2.90–10.1)	4.60 (3.00–7.07)
Generalised anxiety disorder^¶^	3.56 (2.55–4.98)	4.33 (3.18–5.90)	3.94 (3.15–4.94)	2.82 (1.99–3.99)	3.48 (2.53–4.80)	3.14 (2.48–3.97)
Alcohol use disorder — mild^¶^	1.17 (0.93–1.48)	1.71 (1.25–2.35)	1.35 (1.13–1.62)	1.17 (0.92–1.50)	1.59 (1.13–2.23)	1.31 (1.08–1.59)
Alcohol use disorder — moderate^¶^	1.95 (1.32–2.88)	2.48 (1.54–4.00)	2.14 (1.59–2.90)	1.78 (1.19–2.65)	2.17 (1.31–3.61)	1.94 (1.42–2.66)
Alcohol use disorder — severe^¶^	2.55 (1.65–3.93)	5.11 (2.79–9.37)	3.18 (2.24–4.53)	2.11 (1.36–3.26)	4.18 (2.23–7.87)	2.62 (1.83–3.76)
Major depressive disorder**	3.21 (2.51–4.11)	3.76 (2.97–4.77)	3.50 (2.95–4.15)	2.83 (2.20–3.64)	3.52 (2.75–4.51)	3.19 (2.68–3.80)

* Model adjusted for age group.

† Model adjusted for age group and gender.

‡ Model adjusted for age group, socio‐economic status (based on postcode of residence and quintiles of the Index of Relative Socio‐Economic Disadvantage), experience of financial hardship during childhood, and current financial strain.

§ Model adjusted for age group, gender, socio‐economic status (based on postcode of residence and quintiles of the Index of Relative Socio‐Economic Disadvantage), experience of financial hardship during childhood, and current financial strain.

¶ Current.

** Lifetime.

Positive associations were present for all forms of child maltreatment across all diagnoses of mental disorders (Box 4). Although the point estimates were generally greater for the associations between sexual abuse and the different mental disorders, the strengths of these associations did not differ significantly from those for other forms of maltreatment. For example, sexual abuse was associated with an almost 2‐fold increase in the likelihood of PTSD (OR, 1.95; 95% CI, 1.47–2.60), whereas physical abuse had a smaller point estimate for the increase in likelihood of PTSD (OR, 1.59; 95% CI, 1.17–2.18). However, the overlapping CIs mean that the difference in the strengths of these associations was non‐significant.

Box 4 **Odds ratios for mental disorders in Australians 16 years and older, by type of maltreatment**

**Table:** 

	Odds ratio (95% CI) — adjusted for age and gender			Odds ratio (95% CI) — fully adjusted		
	Men*	Women*	Total^†^	Men^‡^	Women^‡^	Total^§^
**Post‐traumatic stress disorder** ^¶^						
Emotional abuse	2.05 (1.26–3.31)	2.01 (1.26–3.21)	2.06 (1.47–2.89)	1.94 (1.18–3.18)	1.96 (1.23–3.12)	1.98 (1.41–2.77)
Neglect	1.78 (1.04–3.03)	2.25 (1.53–3.31)	1.92 (1.41–2.61)	1.76 (1.01–3.08)	1.97 (1.29–3.03)	1.71 (1.22–2.38)
Physical abuse	2.91 (1.72–4.93)	1.13 (0.77–1.68)	1.69 (1.24–2.32)	2.69 (1.62–4.45)	1.06 (0.71–1.57)	1.59 (1.17–2.18)
Sexual abuse	1.89 (1.22–2.92)	2.45 (1.68–3.57)	2.17 (1.65–2.87)	1.68 (1.07–2.63)	2.18 (1.48–3.22)	1.95 (1.47–2.60)
Exposure to domestic violence	1.13 (0.69–1.85)	2.05 (1.34–3.15)	1.60 (1.17–2.20)	1.06 (0.64–1.77)	2.05 (1.31–3.20)	1.53 (1.10–2.14)
**Generalised anxiety disorder** ^¶^						
Emotional abuse	2.08 (1.50–2.88)	2.26 (1.64–3.10)	2.19 (1.74–2.75)	2.00 (1.44–2.79)	2.19 (1.59–3.02)	2.13 (1.69–2.68)
Neglect	1.40 (0.94–2.08)	1.65 (1.18–2.30)	1.53 (1.19–1.96)	1.40 (0.91–2.15)	1.40 (0.98–1.99)	1.35 (1.03–1.77)
Physical abuse	1.94 (1.40–2.68)	1.09 (0.82–1.47)	1.41 (1.14–1.74)	1.80 (1.31–2.47)	1.05 (0.78–1.41)	1.34 (1.08–1.66)
Sexual abuse	1.69 (1.24–2.31)	1.94 (1.50–2.51)	1.81 (1.49–2.19)	1.51 (1.10–2.08)	1.78 (1.37–2.32)	1.65 (1.35–2.01)
Exposure to domestic violence	1.32 (0.95–1.84)	1.30 (0.97–1.75)	1.32 (1.06–1.64)	1.26 (0.90–1.76)	1.25 (0.92–1.70)	1.25 (1.00–1.56)
**Alcohol use disorder — mild** ^**¶**^						
Emotional abuse	0.79 (0.58–1.08)	0.99 (0.70–1.39)	0.87 (0.70–1.10)	0.79 (0.58–1.08)	0.96 (0.68–1.35)	0.86 (0.69–1.08)
Neglect	0.70 (0.41–1.19)	0.74 (0.48–1.15)	0.75 (0.54–1.05)	0.72 (0.41–1.27)	0.71 (0.44–1.16)	0.75 (0.52–1.07)
Physical abuse	1.14 (0.86–1.51)	0.99 (0.70–1.40)	1.08 (0.87–1.35)	1.14 (0.86–1.51)	1.00 (0.71–1.40)	1.08 (0.87–1.35)
Sexual abuse	1.09 (0.80–1.49)	1.55 (1.14–2.11)	1.35 (1.10–1.66)	1.08 (0.79–1.47)	1.53 (1.12–2.10)	1.34 (1.08–1.65)
Exposure to domestic violence	1.07 (0.82–1.39)	1.55 (1.14–2.10)	1.22 (1.00–1.49)	1.05 (0.80–1.37)	1.49 (1.09–2.05)	1.18 (0.97–1.45)
**Alcohol use disorder — moderate** ^**¶**^						
Emotional abuse	1.11 (0.73–1.70)	1.86 (1.14–3.03)	1.38 (1.01–1.90)	1.08 (0.71–1.66)	1.83 (1.12–2.99)	1.35 (0.99–1.85)
Neglect	0.83 (0.43–1.60)	0.84 (0.47–1.50)	0.85 (0.56–1.30)	0.78 (0.39–1.53)	0.69 (0.38–1.24)	0.74 (0.48–1.15)
Physical abuse	1.18 (0.79–1.76)	1.26 (0.81–1.97)	1.21 (0.90–1.63)	1.16 (0.77–1.73)	1.23 (0.79–1.91)	1.19 (0.89–1.61)
Sexual abuse	1.52 (1.02–2.25)	1.71 (1.14–2.56)	1.62 (1.23–2.13)	1.44 (0.95–2.17)	1.65 (1.08–2.54)	1.55 (1.16–2.07)
Exposure to domestic violence	1.46 (0.99–2.16)	1.11 (0.70–1.75)	1.31 (0.98–1.77)	1.41 (0.95–2.08)	1.05 (0.66–1.68)	1.28 (0.95–1.73)
**Alcohol use disorder — severe** ^**¶**^						
Emotional abuse	1.02 (0.68–1.54)	1.78 (1.05–3.03)	1.31 (0.95–1.81)	0.97 (0.64–1.46)	1.72 (1.01–2.94)	1.26 (0.91–1.74)
Neglect	1.27 (0.73–2.20)	1.16 (0.64–2.10)	1.21 (0.81–1.80)	1.24 (0.68–2.24)	1.08 (0.56–2.11)	1.09 (0.70–1.70)
Physical abuse	1.45 (0.96–2.18)	1.01 (0.57–1.78)	1.24 (0.88–1.73)	1.36 (0.92–2.01)	0.95 (0.54–1.68)	1.18 (0.85–1.64)
Sexual abuse	1.69 (1.13–2.52)	3.59 (2.27–5.69)	2.33 (1.75–3.08)	1.56 (1.04–2.35)	3.30 (2.08–5.21)	2.12 (1.59–2.82)
Exposure to domestic violence	1.70 (1.15–2.51)	1.06 (0.59–1.90)	1.43 (1.02–2.00)	1.62 (1.11–2.36)	1.04 (0.57–1.89)	1.36 (0.97–1.90)
**Major depressive disorder** **						
Emotional abuse	2.12 (1.60–2.82)	1.78 (1.38–2.30)	1.92 (1.59–2.32)	2.07 (1.56–2.76)	1.78 (1.37–2.30)	1.90 (1.57–2.30)
Neglect	1.30 (0.89–1.89)	0.84 (0.61–1.14)	0.96 (0.76–1.23)	1.22 (0.83–1.79)	0.88 (0.64–1.22)	0.96 (0.75–1.23)
Physical abuse	1.40 (1.09–1.80)	1.14 (0.89–1.46)	1.23 (1.04–1.47)	1.35 (1.05–1.74)	1.15 (0.90–1.48)	1.23 (1.03–1.46)
Sexual abuse	1.64 (1.27–2.12)	1.71 (1.39–2.11)	1.68 (1.43–1.97)	1.61 (1.24–2.09)	1.72 (1.39–2.13)	1.66 (1.41–1.95)
Exposure to domestic violence	1.38 (1.06–1.78)	1.53 (1.22–1.93)	1.46 (1.23–1.74)	1.32 (1.01–1.71)	1.49 (1.18–1.87)	1.41 (1.19–1.68)

* Model adjusted for age group.

† Model adjusted for age group and gender.

‡ Model adjusted for age group, socio‐economic status (based on postcode of residence and quintiles of the Index of Relative Socio‐Economic Disadvantage), experience of financial hardship during childhood, and current financial strain.

§ Model adjusted for age group, gender, socio‐economic status (based on postcode of residence and quintiles of the Index of Relative Socio‐Economic Disadvantage), experience of financial hardship during childhood, and current financial strain.

¶ Current.

** Lifetime.

Sexual abuse was the only form of child maltreatment associated with all three levels of severity of AUDs. Importantly, emotional abuse was also consistently and independently associated with increased odds of most mental disorders, with associations similar in magnitude to those for sexual and physical abuse (Box 4). There were no significant differences between women and men in the strengths of association between each of the mental disorders and each type of child maltreatment. In all three age groups, there was a positive association between experiences of child maltreatment and the odds of having a mental disorder (Supporting Information, table 2), and the likelihood of having any mental disorder in those who experienced maltreatment was about three times higher than for those who did not experience maltreatment in all age groups. The point estimates were greatest for the association between maltreatment and PTSD in the youngest cohort (16–24 years), although the overlapping CIs suggest that the difference was not significant. The associations between reported child maltreatment and mental disorders persisted throughout life (Supporting Information, table 3).

## Discussion

In this national survey, we measured experiences of all five forms of child maltreatment and diagnoses of mental disorders, and there were five key findings. First, the prevalence of mental disorders was significantly increased for Australians who experienced any child maltreatment compared with that for those who did not experience child maltreatment (48.0% *v* 21.6%), and higher again for those who experienced multi‐type child maltreatment (54.8%). Second, all five forms of maltreatment were consistently associated with a 2–3‐fold increase in the odds of a mental disorder diagnosis across genders and ages. Third, adjusting for current and childhood financial disadvantage did not significantly attenuate these associations, suggesting that the association between child maltreatment and mental disorders is independent of these social determinants of health. Fourth, all forms of child maltreatment were similarly associated with mental health harm, although associations were strongest for sexual abuse and emotional abuse. Fifth, prevalence of common mental disorders such as PTSD and moderate and severe AUD was very low in Australians who had not experienced child maltreatment.

Findings from previous research suggest a causal relationship between child maltreatment and mental disorders.^5^, ^16^ Biological changes and psychosocial challenges often experienced by maltreated children are responsible for the increased risk of mental disorders. Child maltreatment leads to cognitive alterations including distrust of others, hypervigilance to threat, impaired emotion recognition and regulation, and reduced responsiveness to rewards.^17^, ^18^, ^19^ Experiences of child maltreatment heighten threat perception, which activates the body's stress response and sensitises the neurobiological systems, making an individual more vulnerable to mental illness.^20^ In addition, low reward responsiveness, a key element of neglect and punitive parenting, is underpinned by neural changes associated with depression.^21^, ^22^ Experiences of child maltreatment disrupt emotion recognition and regulation skills, which are critical for healthy relationships with peers^19^ and foundational to interpersonal relationships throughout life. These maladaptive interpersonal problems — for example, the premature sexualisation and shame that accompany sexual abuse — compromise the ability of some maltreated children to form stable friendships, which may lead to persistent relationship challenges over the life course.^23^, ^24^ In this way, child maltreatment initiates a developmental cascade that disrupts social connection and other opportunities,^24^ conferring risk of mental disorders. In summary, the increased risk of multiple biological changes and psychosocial challenges in maltreated children are hypothesised to increase the risk of mental illness.

The strengths of the associations between experiences of child maltreatment and mental disorders were similar in all three age groups. The typical persistent course of mental illness may explain the consistency of the relationship between maltreatment and mental disorders throughout life. The focus on service provision rather than prevention has had no impact on the population prevalence of mental disorders in Australia.^25^ Whatever the mechanism by which maltreatment is associated with mental disorders, the prevention of harm to children must be a foundation of any mental health policy addressing mental illness in the population.

Australian mental health strategies have failed to integrate the prevention of child maltreatment with other policy initiatives.^3^ This is important given the high burden of disease associated with depression and anxiety that is directly attributable to child maltreatment.^8^ Child protection is largely siloed from health services, frequently leading to inadequate management of co‐occurring health and social problems in maltreated children.^26^ Improving parenting skills and supporting healthy family interactions are fundamental to prevention of child maltreatment.^27^ Universal implementation of evidence‐based parenting interventions^28^ and targeted delivery of nurse home visiting programs and interventions for mental disorders and substance use disorders to vulnerable parents^29^, ^30^ are critical to make population‐level reductions to the prevalence of child maltreatment and the associated mental health harm. To be most effective, these need to be underpinned by policies ensuring a living minimum wage and welfare support, and affordable and available child care and housing. The provision of evidence‐based mental health interventions that address the harms experienced by maltreated children^31^, ^32^ is required to prevent mental disorders from emerging and persisting throughout life; this has the potential to reduce associated costs and burden on the health care system.

### Limitations

Maltreatment was assessed by retrospective self‐report. It is possible that those living with mental disorders may be biased towards negative memories and, conversely, those without mental disorders may be more likely to minimise maltreatment that occurred in childhood.^33^ To reduce this risk, the Juvenile Victimisation Questionnaire‐R2 adapted version (Australian Child Maltreatment Study) screened for child maltreatment with behaviourally specific items that assessed objective events to maximise the accuracy of participant recall. There may be unmeasured confounders that could attenuate the association between child maltreatment and later mental disorders,^34^ such as parental mental illness.^35^, ^36^ To reduce participant burden, selected modules from the MINI that we used to assess DSM‐5 mental disorder diagnoses included point prevalence for some disorders (PTSD, GAD and AUD) and lifetime prevalence for MDD. The combination of time frames in our study is a limitation in our reporting of combined mental disorder prevalence. It will have led to underestimation of lifetime prevalence data, but has provided an overall estimate of point prevalence. Direct comparisons of prevalence between data from our survey and data collected during the period 2020–2021 in the National Survey of Mental Health and Wellbeing (which used DSM‐IV)^2^ are limited by differences in time frames and diagnostic criteria for measurement of mental disorders. This is particularly salient for mild AUD, which has a low threshold for diagnosis in DSM‐5, requiring only two of the 11 dependence symptoms over the previous 12 months. However, some direct comparisons can be made. For example, the 12‐month prevalence of PTSD in the 2020–2021 National Survey of Mental Health and Wellbeing data was 5.7%, comparable to the 5.3% point prevalence in our study. We only assessed common mental disorders in our study, because the sample was not large enough to diagnose low prevalence disorders such as schizophrenia and bipolar disorder. However, the assessed disorders are responsible for most of the burden of disease attributable to mental disorders in Australia.^1^ As noted elsewhere, few participants identified as gender diverse.^12^ Thus, owing to small cell sizes and important aspects of gender diverse identification such as heterogeneity, we will report detailed analysis of findings for these participants separately. Finally, the study may not have been adequately powered to detect differences between men and women in strengths of associations between child maltreatment and certain mental disorders. It is likely that a larger sample would have shown that the associations between child maltreatment and PTSD were stronger in women than in men.

### Conclusion

The prevalence of mental disorders is substantially higher in those who have experienced any type of child maltreatment and, particularly, multi‐type maltreatment. Mental disorders cause significant suffering that all too frequently persists throughout life. They are responsible for a substantial burden on health care resources and expenditure. Prevention of child maltreatment provides an opportunity to substantially reduce the prevalence of mental disorders and improve the health of the Australian population.

## Open access

Open access publishing facilitated by The University of Queensland, as part of the Wiley ‐ The University of Queensland agreement via the Council of Australian University Librarians.

## Agency roles

The NHMRC funded the ACMS. The Australian Government and the Australian Institute of Criminology provided supplementary funding for several specific questions. The researchers were independent from the funders.

## Competing interests

No relevant disclosures.

## Supporting information


Supporting Information
