# Supplementary material for: The association between child maltreatment and mental disorders in the Australian Child Maltreatment Study
Source: Med J Aust. 2023 Apr 2;218(Suppl 6):S26–33. doi: 10.5694/mja2.51870 (PMC10952950; doi:10.5694/mja2.51870)
Supplement: Supplementary file 1 — Supporting Information [file MJA2-218-S26-s001.pdf]

Supporting Information

Supplementary methods and results

This appendix was part of the submitted manuscript and has been peer reviewed.
It is posted as supplied by the authors.

Appendix to: Scott JG, Malacova E, Mathews B, et al. The association between child maltreatment and mental disorders in the Australian Child Maltreatment Study. *Med J Aust* 2023; doi: 10.5694/mja2.51870.

Supporting Information

Table 1. Maltreatment items as assessed in the Juvenile Victimisation Questionnaire-R2 adapted version (Australian Child Maltreatment Study)

Type of maltreatment	Maltreatment subtype
**Emotional abuse (3 items)**	
Did any of your parents insult you, humiliate you, or call you hurtful names?	Hostile interaction/denigration
Did any of your parents tell you they hated you, didn't love you, wished you were dead or had never been born?	Rejection
Did any of your parents often ignore you, or not show you love and affection?	Emotional unavailability
**Neglect (3 items)**	
Was your home often unsafe or unhealthy? For example, it had toilets or sinks that didn't work, rubbish piled up, and things like that?	Environmental neglect
Were you often not provided with regular meals, baths or showers, or clean clothes?	Nutritional/physical neglect
When you were sick or injured, did your parent ever fail to get you medical care or take care of you?	Medical neglect
**Physical abuse (2 items)**	
Did an adult ever beat you up, hit you on the head or face, choke you, or burn you?	Severe physical abuse
Did an adult ever hit, punch, kick, or physically hurt you?	Moderate physical abuse
**Sexual abuse (4 items)**	
Did anyone ever look at your private parts when they shouldn't have, or make you look at their private parts?	Abusive exposure
Did anyone ever touch your private parts when they shouldn't have, or make you touch their private parts?	Abusive touching (contact abuse short of intercourse)
Did anyone ever try to force you to have sex, even if it didn't happen?	Attempted intercourse
Did anyone ever force you to have sex?	Abusive intercourse
**Exposure to domestic violence (4 items)**	
Did you ever see or hear one of your parents get pushed, hit, choked, or beaten up by your other parent or their partner?	Exposure to physical violence between parents
Did you ever see or hear one of your parents seriously threaten to hurt your other parent?	Exposure to serious threats of domestic violence
During an argument, did any of your parents ever damage any property or pets, punch the wall, or throw something?	Exposure to damage of property or pets
Did you ever see or hear one of your parents intimidate or control your other parent, either verbally, sexually, financially, or by isolating them from friends or family?	Exposure to intimidation or control

Table 2. Proportion of adults with mental disorders, with experience of child maltreatment, by gender and age group

Experience of child maltreatment	Participants with mental disorders — *N* = 3606; percentage (95% CI)								
	16–24-year-olds (*n* = 1749)			25–44-year-olds (*n* = 909)			≥ 45-year-olds (*n* = 948)		
	Men	Women	Total#	Men	Women	Total#	Men	Women	Total#
**Any mental disorder**									
No	264; 30.1% (26.8–33.5%)	178; 29.2% (25.2–33.2%)	447; 29.8% (27.2–32.4%)	112; 29.2% (24.2–34.2%)	76; 23.5% (18.4–28.7%)	189; 26.6% (23.0–30.2%)	133; 20.9% (17.4–24.4%)	81; 12.2% (9.4–15.1%)	215; 16.6% (14.3–18.9%)
Yes	536; 54.4% (50.9–58.0%)	704; 63.8% (60.7–67.0%)	1302; 60.0% (57.7–62.4%)	335; 57.0% (52.7–61.4%)	374; 55.4% (51.3–59.5%)	720; 56.2% (53.3–59.2%)	319; 37.5% (33.9–41.2%)	411; 38.9% (35.5–42.3%)	733; 38.3% (35.8–40.8%)
**Post-traumatic stress disorder***									
No	8; 1.0% (0.3–1.7%)	11; 1.9% (0.7–3.0%)	20; 1.4% (0.8–2.0%)	5; 1.8% (0.2–3.4%)	np	8; 1.4% (0.4–2.4%)	7; 1.1% (0.2–2.0%)	6; 1.2% (0.2–2.2%)	13; 1.1% (0.5–1.8%)
Yes	62; 6.0% (4.5–7.6%)	157; 15.5% (13.1–17.9%)	236; 11.5% (10.0–13.0%)	43; 7.5% (5.2–9.8%)	59; 9.3% (6.9–11.7%)	108; 8.7% (7.0–10.4%)	42; 6.2% (4.2–8.1%)	60; 6.0% (4.4–7.7%)	103; 6.1% (4.8–7.4%)
**Generalised anxiety disorder***									
No	37; 4.5% (3.0–6.1%)	62; 10.7% (8.0–13.4%)	100; 7.2% (5.8–8.7%)	21; 5.9% (3.2–8.6%)	20; 7.2% (3.9–10.5%)	42; 6.5% (4.5–8.6%)	18; 2.9% (1.4–4.3%)	14; 1.8% (0.7–2.8%)	33; 2.4% (1.5–3.3%)
Yes	170; 18.0% (15.2–20.8%)	331; 30.5% (27.5–33.5%)	546; 25.8% (23.7–27.8%)	100; 17.7% (14.3–21.1%)	131; 20.4% (17.1–23.8%)	240; 19.6% (17.2–22.0%)	73; 9.4% (7.2–11.7%)	113; 11.9% (9.6–14.2%)	187; 10.8% (9.2–12.4%)
**Alcohol use disorder — mild***									
No	124; 14.4% (11.8–16.9%)	53; 8.2% (5.9–10.5%)	177; 11.6% (9.9–13.4%)	55; 14.0% (10.2–17.9%)	23; 7.1% (4.0–10.2%)	78; 10.9% (8.4–13.5%)	64; 10.1% (7.5–12.7%)	26; 4.0% (2.3–5.8%)	90; 7.1% (5.5–8.7%)
Yes	173; 16.6% (14.1–19.1%)	170; 14.9% (12.6–17.1%)	352; 15.5% (13.9–17.2%)	109; 18.7% (15.2–22.2%)	77; 11.2% (8.6–13.8%)	187; 14.6% (12.4–16.7%)	94; 10.5% (8.3–12.7%)	79; 6.9% (5.2–8.6%)	174; 8.6% (7.2–9.9%)

**Alcohol use disorder — moderate***									
No	47; 4.8% (3.4–6.2%)	19; 3.2% (1.7–4.7%)	66; 4.1% (3.1–5.1%)	17; 5.6% (2.8–8.4%)	6; 1.9% (0.3–3.6%)	23; 4.0% (2.2–5.7%)	12; 2.1% (0.8–3.4%)	11; 1.6% (0.6–2.6%)	23; 1.8% (1.0–2.6%)
Yes	89; 8.2% (6.5–9.9%)	90; 7.4% (5.8–9.0%)	185; 7.7% (6.6–8.9%)	47; 7.6% (5.3–9.9%)	36; 4.8% (3.1–6.5%)	84; 6.1% (4.7–7.5%)	55; 6.6% (4.7–8.4%)	49; 4.1% (2.8–5.4%)	105; 5.2% (4.1–6.3%)
**Alcohol use disorder — severe***									
No	18; 1.8% (0.9–2.7%)	10; 1.5% (0.6–2.5%)	28; 1.7% (1.0–2.4%)	16; 4.7% (2.3–7.1%)	5; 1.4% (0.1–2.7%)	21; 3.2% (1.8–4.7%)	9; 2.0% (0.6–3.3%)	np	13; 1.3% (0.5–2.0%)
Yes	80; 8.8% (6.7–10.9%)	72; 6.6% (5.0–8.3%)	162; 7.9% (6.6–9.2%)	57; 10.1% (7.3–12.8%)	47; 8.1% (5.7–10.4%)	106; 9.0% (7.2–10.8%)	40; 4.9% (3.3–6.6%)	26; 2.4% (1.3–3.4%)	66; 3.5% (2.6–4.4%)
**Major depressive disorder†**									
No	88; 10.1% (7.9–12.3%)	78; 12.5% (9.6–15.4%)	169; 11.2% (9.5–13.0%)	33; 8.3% (5.3–11.2%)	42; 12.6% (8.7–16.5%)	75; 10.1% (7.7–12.5%)	44; 6.1% (4.1–8.0%)	42; 6.2% (4.1–8.2%)	86; 6.1% (4.7–7.5%)
Yes	229; 23.3% (20.3–26.3%)	352; 32.1% (29.1–35.2%)	601; 27.9% (25.8–30.0%)	152; 25.1% (21.3–28.9%)	218; 31.3% (27.5–35.1%)	375; 28.3% (25.6–30.9%)	152; 17.3% (14.5–20.0%)	257; 24.0% (21.1–27.0%)	410; 21.0% (18.9–23.0%)

Includes participants who identified as gender diverse; np = not published because of small cell size. * Current. † Lifetime.

Table 3. Odds of having a mental disorder in participants exposed to maltreatment, stratified by gender and age group

	Odds ratio (95% CI) — adjusted for age and gender			Odds ratio (95% CI) — fully adjusted		
	**Men***	**Women***	**Total†**	**Men‡**	**Women‡**	**Total§**
**16–24-year-olds**						
Any mental disorder	2.77 (2.23–3.43)	4.28 (3.37–5.44)	3.41 (2.91–3.99)	2.36 (1.88–2.97)	3.44 (2.68–4.43)	2.85 (2.41–3.36)
Post-traumatic stress disorder¶	6.30 (2.90–13.6)	9.60 (5.00–18.4)	8.20 (5.00–13.3)	4.51 (1.90–10.5)	6.68 (3.40–12.9)	5.83 (3.52–9.65)
Generalised anxiety disorder¶	4.63 (3.11–6.90)	3.65 (2.66–5.02)	4.05 (3.17–5.19)	3.85 (2.53–5.88)	2.91 (2.07–4.09)	3.30 (2.55–4.28)
Alcohol use disorder — mild¶	1.18 (0.90–1.56)	1.95 (1.37–2.77)	1.45 (1.18–1.79)	1.15 (0.86–1.54)	1.89 (1.30–2.74)	1.40 (1.12–1.75)
Alcohol use disorder — moderate¶	1.77 (1.20–2.61)	2.40 (1.41–4.10)	2.01 (1.48–2.74)	1.57 (1.04–2.37)	2.39 (1.37–4.17)	1.89 (1.36–2.61)
Alcohol use disorder — severe¶	5.15 (2.93–9.04)	4.52 (2.27–9.01)	4.96 (3.21–7.67)	4.46 (2.42–8.21)	3.46 (1.67–7.18)	4.05 (2.54–6.47)
Major depressive disorder**	2.72 (2.02–3.65)	3.31 (2.45–4.47)	2.96 (2.41–3.65)	2.51 (1.84–3.43)	2.99 (2.16–4.12)	2.72 (2.18–3.39)
**25–44-year-olds**						
Any mental disorder	3.22 (2.38–4.35)	4.04 (2.90–5.63)	3.58 (2.87–4.47)	2.66 (1.94–3.65)	3.54 (2.49–5.02)	3.02 (2.39–3.81)
Post-traumatic stress disorder¶	4.50 (1.7–11.7)	10.8 (3.20–35.8)	6.50 (3.10–13.8)	3.48 (1.28–9.50)	8.78 (2.50–30.8)	5.09 (2.30–11.1)
Generalised anxiety disorder¶	3.42 (2.00–5.84)	3.30 (1.93–5.64)	3.40 (2.33–4.95)	2.67 (1.51–4.71)	2.53 (1.45–4.43)	2.63 (1.78–3.90)
Alcohol use disorder — mild¶	1.41 (0.95–2.08)	1.65 (0.96–2.85)	1.49 (1.09–2.04)	1.39 (0.92–2.11)	1.68 (0.93–3.03)	1.50 (1.07–2.10)
Alcohol use disorder — moderate¶	1.39 (0.75–2.59)	2.55 (0.98–6.66)	1.67 (1.00–2.78)	1.42 (0.76–2.63)	2.03 (0.70–5.88)	1.55 (0.91–2.64)
Alcohol use disorder — severe¶	2.27 (1.22–4.21)	6.14 (2.30–16.30)	3.07 (1.84–5.12)	1.67 (0.87–3.23)	4.83 (1.70–13.7)	2.38 (1.38–4.09)
Major depressive disorder**	3.72 (2.39–5.79)	3.16 (2.12–4.71)	3.43 (2.55–4.61)	3.08 (1.94–4.89)	3.05 (2.00–4.65)	3.10 (2.28–4.22)

**≥ 45-year-olds**						
Any mental disorder	2.28 (1.75–2.97)	4.57 (3.38–6.19)	3.14 (2.58–3.82)	2.08 (1.58–2.73)	3.94 (2.87–5.41)	2.79 (2.27–3.42)
Post-traumatic stress disorder¶	5.90 (2.50–14.3)	5.40 (2.10–13.6)	5.70 (3.00–10.8)	4.38 (1.80–10.8)	3.88 (1.50–10.3)	4.13 (2.14–7.97)
Generalised anxiety disorder¶	3.54 (1.99–6.32)	7.6 (4.0–14.3)	4.93 (3.24–7.50)	2.75 (1.52–4.98)	6.15 (3.20–11.9)	3.94 (2.55–6.09)
Alcohol use disorder — mild¶	1.04 (0.72–1.52)	1.78 (1.05–3.01)	1.28 (0.95–1.72)	1.08 (0.73–1.60)	1.48 (0.82–2.67)	1.21 (0.88–1.66)
Alcohol use disorder — moderate¶	3.32 (1.62–6.77)	2.68 (1.30–5.54)	3.06 (1.83–5.10)	2.79 (1.31–5.93)	2.32 (1.07–4.99)	2.63 (1.53–4.53)
Alcohol use disorder — severe¶	2.56 (1.18–5.54)	4.40 (1.40–13.8)	2.97 (1.56–5.64)	2.39 (1.16–4.93)	3.56 (1.20–10.9)	2.72 (1.47–5.02)
Major depressive disorder**	3.23 (2.18–4.78)	4.81 (3.27–7.09)	4.02 (3.06–5.30)	2.94 (1.97–4.38)	4.45 (2.98–6.65)	3.70 (2.79–4.91)

* Model unadjusted. † Model adjusted for gender. ‡ Model adjusted for socioeconomic status (based on postcode of residence and quintiles of the Index of Relative Socio-Economic Disadvantage), experience of financial hardship during childhood and current financial strain. § Model adjusted for gender, socioeconomic status (based on postcode of residence and quintiles of the Index of Relative Socio-Economic Disadvantage), experience of financial hardship during childhood and current financial strain. ¶ Current. ** Lifetime.
